# Current and Future Perspectives on the Role of Probiotics, Prebiotics, and Synbiotics in Controlling Pathogenic *Cronobacter* Spp. in Infants

**DOI:** 10.3389/fmicb.2021.755083

**Published:** 2021-10-21

**Authors:** Alfred Ke, Valeria R. Parreira, Lawrence Goodridge, Jeffrey M. Farber

**Affiliations:** Department of Food Science, Canadian Research Institute for Food Safety, University of Guelph, Guelph, ON, Canada

**Keywords:** *Cronobacter*, probiotics, prebiotics, synbiotics, short-chain fatty acids, cell-free supernatant, gut model

## Abstract

*Cronobacter* species, in particular *C. sakazakii*, is an opportunistic bacterial pathogen implicated in the development of potentially debilitating illnesses in infants (<12months old). The combination of a poorly developed immune system and gut microbiota put infants at a higher risk of infection compared to other age groups. Probiotics and prebiotics are incorporated in powdered infant formula and, in addition to strengthening gut physiology and stimulating the growth of commensal gut microbiota, have proven antimicrobial capabilities. Postbiotics in the cell-free supernatant of a microbial culture are derived from probiotics and can also exert health benefits. Synbiotics, a mixture of probiotics and prebiotics, may provide further advantages as probiotics and gut commensals degrade prebiotics into short-chain fatty acids that can provide benefits to the host. Cell-culture and animal models have been widely used to study foodborne pathogens, but sophisticated gut models have been recently developed to better mimic the gut conditions, thus giving a more accurate representation of how various treatments can affect the survival and pathogenicity of foodborne pathogens. This review aims to summarize the current understanding on the connection between *Cronobacter* infections and infants, as well as highlight the potential efficacy of probiotics, prebiotics, and synbiotics in reducing invasive *Cronobacter* infections during early infancy.

## Introduction

*Cronobacter sakazakii* has been implicated in the development of neonatal infections including necrotizing enterocolitis (NEC), bacteremia, and meningitis with mortality rates ranging from 40 to 80% for premature (<37weeks gestational age) and/or low-birthweight infants (<2,500g) (Bowen and Braden, 2006; Ray et al., 2007; Friedemann, 2009; Centre for Disease Control, 2020a), and the survivors of *C. sakazakii* infection may develop chronic sequelae such as neurological impairments (Holý et al., 2019). Powdered infant formula (PIF) is a common source of *C. sakazakii* infection, but the pathogen can be found in hospitals, homes, and equipment, e.g., enteral feeding tube and baby bottles (Kalyantanda et al., 2015; Centre for Disease Control, 2020b). *Cronobacter sakazakii* contamination rates of 3–7 and 5% from PIF or PIF processing plants, respectively, have been found (Mardaneh and Dallal, 2016; Fei et al., 2017a; Lu et al., 2019). Recommendations from the World Health Organization (WHO) to minimize contamination of PIF, such as using water >70°C to rehydrate PIF or exclusively breastfeeding infants up to 6months old (World Health Organization, 2007), has not been shown to eliminate the risk of *C. sakazakii* infection as outbreaks continue to occur, so other preventative strategies are needed.

The infant gut microbiota matures in the first 3years of life, and its composition is affected by many factors including childbirth delivery method, infant diet, gestational age, maternal diet, environment, and genetics (Yatsunenko et al., 2012; Bäckhed et al., 2015; Milani et al., 2017). The infant gut microbiota hosts a few key genera associated with a healthy gut, such as *Bifidobacterium* and *Lactobacillus*, which dominate the gut microbiota of 1-year-old breast-fed infants (Bäckhed et al., 2015). PIF poses a greater risk of *C. sakazakii* infection in infants and cannot establish a healthy gut microbiota as compared to breast-fed infants (Le Doare et al., 2018). Breast milk is estimated to contribute approximately 28% of the infant gut microbiota in an infant’s first 12months of life and contains human milk oligosaccharides (HMOs) that promote a healthy and diverse infant gut microbiota (Mueller et al., 2015; Pannaraj et al., 2017; Le Doare et al., 2018; Robertson et al., 2019). The combination of a diverse gut microbiota and HMOs reduces the risk of illness in infants until the maturation of their gut microbiota occurs following the ingestion of solid foods (Johnson-Henry et al., 2016; Le Doare et al., 2018; Robertson et al., 2019). PIF may be supplemented with probiotics, prebiotics, or a combination of both to partially simulate the complex composition of human breast milk (Ackerberg et al., 2012; Vandenplas et al., 2015), which is a complicated task due to the diverse microbiota and oligosaccharides present. However, current research is being conducted to achieve an ideal cocktail of probiotics and prebiotics that mimics breast milk.

Probiotics have a long history of research and usage, with early recognition and guidance being given by the Food and Agriculture Organization of the United Nations and the World Health organization (FAO/WHO) in 2001/2002 (Food and Agriculture Organization of the United Nations/World Health Organization, 2002), and have demonstrated their ability to inhibit pathogens through direct or indirect antagonism (Mohsin et al., 2015; Tomar et al., 2015; Fijan, 2016). Probiotics produce organic acids, such as lactic acid and acetic acid, and hydrogen peroxide (H_2_O_2_), and bacteriocins that can inhibit foodborne pathogens such as *Listeria monocytogenes*, *Escherichia coli*, and *Salmonella* spp. (Makras and De Vuyst, 2006; Kotsou et al., 2008). Furthermore, probiotic strains can release metabolites into the growth medium, collectively referred to as postbiotics, which could play a role in conferring beneficial effects on the host (Tomar et al., 2015). Prebiotics, on the other hand, are predominantly oligosaccharides that contain at least three monomeric units and are neither digested nor absorbed in the small intestine (Venegas et al., 2019). Short-chain fatty acids (SCFAs), for example, butyrate, acetate, and propionate, are produced primarily in the colon by the gut microbiota that metabolizes prebiotics and are considered to be a sub-category of organic acids (Gibson et al., 2017). Acetate and propionate are mainly produced by the Bacteroidetes phyla, while the Firmicute phyla mostly produce butyrate. Bifidobacteria and lactic acid bacteria, for example, can ferment carbohydrates to produce acetate and lactate (Korakli et al., 2002). However, other members of the gut microbiota can act as secondary fermenters to produce other SCFAs (Venegas et al., 2019). SCFAs play an important role in host health by regulating metabolic activities, the immune system, gut epithelial integrity, and by exhibiting antimicrobial properties (Gibson et al., 2017; Robertson et al., 2019). Probiotics and prebiotics have been used to modulate a naïve gut microbiota, but synbiotics have been recommended as an alternative approach to capitalize on their synergism. The concept of synbiotics was introduced by Gibson and Roberfroid (1995) as a mixture of probiotics and prebiotics that improve the survival and colonization of beneficial microbes in the gastrointestinal (GI) tract by selectively stimulating the growth and/or activation of health promoting bacteria and subsequently improving host health. The synergistic effect of prebiotics and probiotics can be found naturally, e.g., breast milk contains prebiotic oligosaccharides and beneficial bacteria that help to stimulate the development of a healthy infant gut (Le doare et al., 2018; Robertson et al., 2019).

Mammalian cell lines are typically used to study the interactions between pathogens, probiotics, and prebiotics, although synbiotics have rarely been studied in this medium (Feeney et al., 2014; Kim et al., 2015; Tomar et al., 2015; Ye et al., 2016; Holý et al., 2019; Jamwal et al., 2019). While useful to visualize the effects of pathogens or probiotics on live cells, cell lines do not closely represent the complex interaction that occurs within the human intestinal environment. Studies on bacterial pathogenesis *in vivo* using animal models can be challenging to conduct due to ethical, cost, and infrastructure issues. Differences between the animal and human gut have led to the emergence of various *in vitro* gut models, for example, the Simulator of the Human Intestinal Microbial Ecosystem (SHIME) that provides an opportunity to analyze the pathogen-host and pathogen-gut microbiota interactions in more detail (Van den Abbeele et al., 2010; Van de Wiele et al., 2015).

While the antimicrobial and gut-enhancing properties of probiotics and prebiotics have been frequently studied (Tomar et al., 2015; Fijan, 2016), there is a lack of understanding on the role and advantages of synbiotics. Furthermore, the effects of prebiotics and synbiotics on pathogenic *Cronobacter* spp. have not been studied and, compared to other enteropathogens, there are few studies into the efficacy of probiotics to reduce *Cronobacter* (Awaisheh et al., 2013; Weng et al., 2014; Charchoghlyan et al., 2016; Campana et al., 2019; Fan et al., 2019; Jamwal et al., 2019). This review examines (i) the current understanding on the association between pathogenic *Cronobacter* spp. and infants and (ii) the potential for probiotics, prebiotics, and synbiotics to reduce the morbidity and mortality of invasive *Cronobacter* infections during early infancy.

## *Cronobacter* Species

### Taxonomy and Identification of *Cronobacter* Species

*Cronobacter* spp., formerly known as *Enterobacter sakazakii*, are Gram-negative, rod shaped, motile, opportunistic pathogens initially associated with debilitating infections in infants (Yan et al., 2012). The designated name of *Enterobacter sakazakii* encompassed all the members of the *Cronobacter* genus up until 2007 due to limited genomic knowledge (Forsythe, 2018) and *E. sakazakii* was split into 16 biogroups based on phenotype (Farmer III et al., 1980; Iversen et al., 2006). Eventually, multi-locus sequence typing (MLST) was used to further characterize and differentiate different species of *Cronobacter* (Baldwin et al., 2009).

### *Cronobacter* Clinical Relevance and Sources

*Cronobacter* spp. are grouped based on their clinical relevance as shown on Table 1. Among the seven *Cronobacter* spp., *C. sakazakii* is the predominant species causing human illness (Sonbol et al., 2013; Fei et al., 2017b). Based on MLST, sequence type profile 4 (ST4) and ST1 strains of *C. sakazakii* are most often isolated from PIF and hospitalized patients (Joseph and Forsythe, 2011; Joseph et al., 2012). *Cronobacter malonaticus* was initially classified as a subspecies of *C. sakazakii* due to their genetic similarities but has since been differentiated from *C. sakazakii* due to the presence of unique genes required for malonate metabolism (Joseph et al., 2012). More recently, like *C. sakazakii*, *C. malonaticus* was found to cause infection in infants by invading and damaging human gut epithelial cells (Alsonosi et al., 2018). However, *C. sakazakii* has the most open reading frames compared to other *Cronobacter* spp., potentially indicating a higher probability for *C. sakazakii* to encode proteins relevant to stress resistance or pathogenicity (Joseph et al., 2012).

**Table 1:** Clinical relevance of *Cronobacter* species (adapted from Forsythe, 2018).

Group	Species	Relevance
1	*C. sakazakii*, *C. malonaticus*	Major clinically relevant isolates in every age group
2	*C. turicensis*, *C. universalis*	Rarely reported, but *C. turicensis* has been isolated from infants (Fei et al., 2017b), which indicates that it may be clinically relevant
Other	*C. dublinensis*, *C. muytjensii*, *C. condimenti*	Unlikely, but possible, to cause infection

*Cronobacter sakazakii* is ubiquitous and has been isolated from a wide variety of sources including dried herbs and spices, soil, starches, milk products, and expressed breast milk (Berhilevych and Kasianchuk, 2017; Forsythe, 2018; Jang et al., 2018a). However, *C. sakazakii* can be found in PIF, factories, hospitals, and homes (Centre for Disease Control, 2020b). PIF may be contaminated with *C. sakazakii* due to poor hygiene in the PIF production facilities and workflow, such as improper filtration in the spray drying process (Jacobs et al., 2011; Li et al., 2014), but *C. sakazakii* has been found in hospital air, dust, and within the human body, so careful handling of PIF may not eliminate the risk of infection (Forsythe, 2018). Recently, the genome sequences of *C. sakazakii* strains have been obtained from various food products, some of which are low-moisture foods of plant origin such as grains, carrots, and mushroom (Jang et al., 2018b). Elkhawaga et al. (2020) found that approximately 9% of the herbs they tested were contaminated with *C. sakazakii*, which implies a potentially higher risk of *C. sakazakii* illness occurring in parts of the world, where the use of alternative medicine relies on natural ingredients derived from herbs and other plant products, especially tea leaves, licorice, anise, allspice, and oregano (Jang et al., 2018a; Elkhawaga et al., 2020).

### Epidemiology of *Cronobacter sakazakii*

Outbreaks of *C. sakazakii* are infrequent and most are associated with infants (Table 2). In the United States, the incidence rate of *Cronobacter* spp. for adults ≥80years old and 70–79years old is 3.93 cases (per 100,000 population) and 2.11 cases, respectively, compared to the 1.81 cases for infants <1year old (Patrick et al., 2014). However, infections in adults are rarer and often occur with mild symptoms unless the individual has underlying health conditions (Tsai et al., 2013; Yong et al., 2018). As *C. sakazakii* is widespread, the sources of contamination can vary, and reservoirs are unclear. In one outbreak involving high school students, for example, *C. sakazakii* was isolated from leftover cafeteria food and resulted in acute gastroenteritis in 156 individuals (Yong et al., 2018). Overall, the sources of *C. sakazakii* infection in adults and the elderly are unclear as the foodborne pathogen can be found in a wide range of environmental sources including plant and animal-derived foods, sewer water, and dust (Tsai et al., 2013; Yong et al., 2018; Ohira et al., 2021).

**Table 2:** Summary of select global outbreaks of *C. sakazakii* occurring between 1958 and 2016.

Location	Year	Number of cases	Outcome	Patient demographic^a^	References
England	1958	2	Death	Infants	Henry and Fouladkhah, 2019
Greece	1984	11	Sepsis, meningitis, and four deaths	Infants	Arseni et al., 1987
France	1994	13	Nine recovered and four deaths	Infants	Caubilla-Barron et al., 2007
Belgium	1998	12	10 recovered and two deaths	Infants	Van Acker et al., 2001
Israel	1999–2000	2	Meningitis in one infant, but both recovered	Infants	Block et al., 2002
USA	2003	6	Recovered, but limited information	Infants	Bowen and Braden, 2006
Mexico	2010	2	Recovered	Infants	Jackson et al., 2015
United States	2011	4	Recovered with one death	Infants	Andrews, 2011
China	2016	156	Acute gastrointestinal illness	High school students	Yong et al., 2018

a *The age of the affected infants ranged from 5days to 5months old*.

PIF is a major source of *C. sakazakii* infection for infants (Centre for Disease Control, 2020b) because heat-sensitive ingredients (e.g., vitamins) are added after milk sterilization and contamination may occur from the manufacturing environment (Kalyantanda et al., 2015). Once contaminated PIF is packaged, C. *sakazakii* can remain viable for up to 2years (Hu et al., 2018; Parra-Flores et al., 2018a). Furthermore, using water of <70°C to rehydrate PIF might exacerbate the likelihood of contamination (World Health Organization, 2007). Improper handling and/or lack of awareness on how to properly reconstitute PIF can contribute to the survival or contamination of PIF with *C. sakazakii*. It is recommended that reconstituted PIF be used within 2h of preparation and be discarded if it is not finished within a single feeding (World Health Organization, 2007).

Infants, especially those that are <2months old, of low-birthweight (<1,500–2,500g) and/or born prematurely (<37weeks gestational age), are the main age group associated with *C. sakazakii* outbreaks (Lai, 2001; Centre for Disease Control, 2020b). Young children up to 3years of age and low-birthweight or premature infants may be afflicted with dysbiosis (Yang et al., 2016), which is described as an imbalanced gut microbiota that has been associated with a range of illnesses including Crohn’s disease, irritable bowel syndrome, and colitis (Biedermann and Rogler, 2015). Infants born *via* C-section may have diminished transmission from mother-to-infant of key birth canal microbes, such as *Bacteroides* and *Bifidobacterium* (Bäckhed et al., 2015), which could increase the infant’s risk of infection caused by pathogens in food and the environment (Durack and Lynch, 2019). Similarly, formula-fed infants can exhibit the delayed establishment of *Bifidobacterium* and *Lactobacillus* spp. in the gut, thereby increasing the potential risk of colonization by opportunistic pathogens (Collado et al., 2015).

In the early 2000s, FAO and WHO collaborated on a risk assessment for the microbiological safety of PIF. *Salmonella* and *Cronobacter* spp. were classified as Category A pathogens to indicate a clear evidence of causality in PIF contamination (Food and Agriculture Organization of the United Nations/World Health Organization, 2006, 2008). Category B pathogens, including *Escherichia*, *Klebsiella*, and *Citrobacter* genera indicated the plausibility of contamination, while Category C pathogens, such as *Clostridium*, *Staphylococcus*, and *Listeria* genera, were not implicated (Food and Agriculture Organization of the United Nations/World Health Organization, 2006). A risk assessment model for *C. sakazakii* in PIF was created to quantify the specific risks of *C. sakazakii* to infants.^1^ The FAO/WHO model for determining the potential PIF risk in a specific situation is considered as the initial *C. sakazakii* population, in addition to reconstitution temperature, handling methods, and feeding period.

### Pathogenesis of *Cronobacter sakazakii*

**Figure 1:**
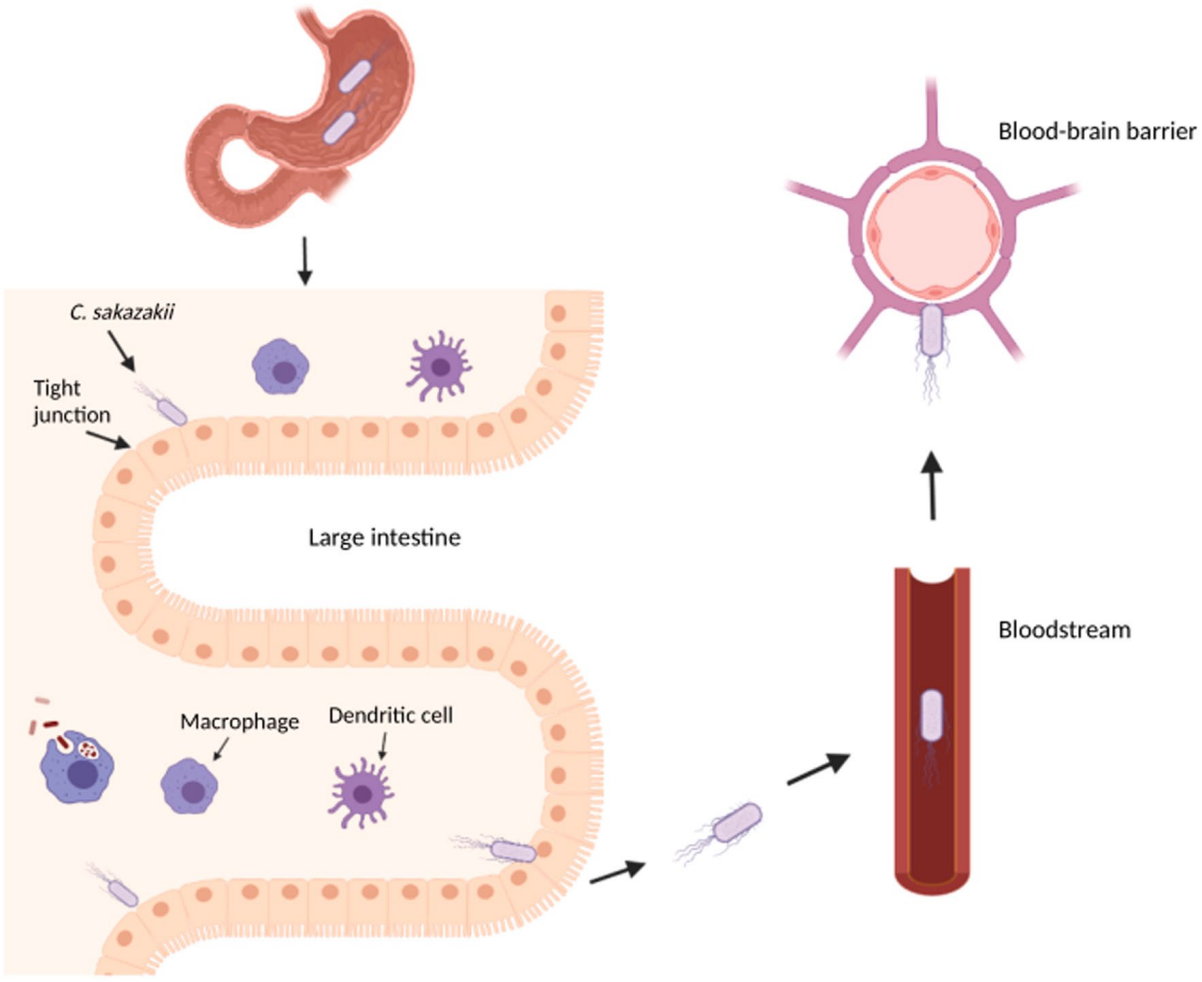
Overview of *Cronobacter sakazakii* pathogenesis in an infant. This diagram illustrates the transit of *C. sakazakii* through the stomach and small intestine to reach the large intestine. In the large intestine, *C. sakazakii* can evade the immune cells to disrupt the epithelial layer, potentially causing necrotizing enterocolitis and entering the bloodstream to cause sepsis. From the bloodstream, *C. sakazakii* can enter the blood–brain barrier to cause meningitis. The image was created with BioRender.com.

The combination of an underdeveloped immune system and an immature intestinal epithelial barrier has been shown to predispose some infants to colonization and infection of pathogens (Emami et al., 2012). In fact, the infection and subsequent development of necrotizing enterocolitis (NEC), a disease that develops when the tissue in the inner lining of the small or large intestine becomes damaged, inflamed, and necrosized, has been linked to the colonization of an infant’s immature gut by *C. sakazakii* (Emami et al., 2012; Figure 1). *Cronobacter sakazakii* can not only suppress the maturation of dendritic cells (DC) but is also able to enter DC to survive and spread, thus avoiding the immune system (Emami et al., 2012). Macrophages and neutrophils have been found to play an important role in eliminating *C. sakazakii* by regulating the production of DC but provide an ineffective immune response due to the intracellular nature of *C. sakazakii* infection (Townsend et al., 2007; Emami et al., 2012). NEC can develop due to pro-inflammatory cytokines released during infection, which stimulates the production of nitric oxide synthase and damages intestinal mucosa through nitric oxide (NO) (Emami et al., 2011). Further disruption of the intestinal layers may allow *C. sakazakii* to reach the brain and/or bloodstream, which can cause meningitis or sepsis, respectively.

### Virulence Factors

Hfq, also known as Host Factor 1, is a regulatory protein involved in the pathogenesis, communication, and survival of several foodborne pathogens including *L. monocytogenes*, *E. coli*, and *Salmonella* Typhimurium (Kim et al., 2015; Cech et al., 2016). The *hfq* gene in *C. sakazakii* was reported to regulate virulence genes, and its absence reduced the infection rate in Caco-2 cells, survival in macrophage-like cells, and oxidative stress resistance (Kim et al., 2015). During desiccation and acid stress conditions, *C. sakazakii* have an increased expression of *hfq* by 1 and 3-logs, respectively (Jameelah et al., 2018; Maerani et al., 2020). However, as *C. sakazakii* entered a viable but not culturable (VBNC) state after desiccation or acid stress conditions, the expression of *hfq* decreased by approximately 1-log (Jameelah et al., 2018; Maerani et al., 2020).

Outer membrane proteins (OMPs) play a significant role in the virulence of *C. sakazakii*. The two OMPs, OmpA and OmpX, were found to be essential for *C. sakazakii* adherence and invasion of intestinal cells (Kim et al., 2015; Singh et al., 2015; Jameelah et al., 2018). The OmpA has also been reported to play a role in facilitating *C. sakazakii* invasion of the blood–brain barrier (Holý et al., 2019). On the other hand, *ompA* or *ompX C. sakazakii* deletion mutants had significantly reduced invasion and adherence rates compared to the wild-type strains (Feeney et al., 2014). The OmpA was also identified as a key protein mediating cell adhesion to fibronectin, a glycoprotein on the extracellular matrix of eukaryotic tissue (Nair et al., 2009).

The enterotoxins and endotoxins from *C*. *sakazakii* remain functional during the manufacturing and shelf life of PIF, which may play a role in the pathogenesis of *C. sakazakii* infection (Jaradat et al., 2014). *Cronobacter sakazakii* enterotoxins may be similar in function to a lipopolysaccharide by stimulating an inflammatory response in the host, which can lead to NEC (Singh et al., 2015), whereas their endotoxins may be able to help to translocate the pathogen across the intestinal and blood–brain barriers to cause bacteremia and meningitis (Kalyantanda et al., 2015; Shi et al., 2017). *Cronobacter sakazakii* has many genes related to survival and virulence, but the function of some of them is still under investigation (Table 3).

**Table 3:** Characteristics of *C. sakazakii* putative virulence and stress genes.

Gene	Encoded product	Putative role	References
*cpa*	Outer membrane protease	Resistance against human serum	Singh et al., 2015; Holý et al., 2019
*dps*	DNA protection	Improves oxidative stress resistance and virulence	Ye et al., 2016; Aly et al., 2019
*fliC*	Flagella assembly	Adhesion to host cells	Aly et al., 2019; Holý et al., 2019
*hfq*	RNA chaperone	Regulation of gene expression and pathogenesis	Kim et al., 2015; Jameelah et al., 2018; Zhang et al., 2019
*hly*	Type III hemolysin	OMP with hemolytic activity	Singh et al., 2015; Holý et al., 2019
*luxs*	Quorum sensing	Enhances virulence and biofilm formation	Ye et al., 2016; Aly et al., 2019
*nanAKTR* operon	Sialic acid metabolism	Pathogenesis and survival in PIF and breast milk	Singh et al., 2015; Aly et al., 2019
*ompA/ompX/ompW*	OMPs	Invasion of human intestinal cells; desiccation resistance	Zhang et al., 2019
*rpoS*	Stress response	Improves cell resistance to stresses; Role in VBNC formation	Jameelah et al., 2018; Zhang et al., 2019
*zpx*	Zinc-containing metalloprotease	Lysis of collagen and distribution outside the GI tract	Joseph et al., 2012; Feeney et al., 2014; Kim et al., 2015

Sialic acid is found in breast milk, PIF, intestinal mucin, and human brain, as it is a bioactive compound needed for brain development (Joseph et al., 2013; Kalyantanda et al., 2015; Lis-Kuberka and Orczyk-Pawiłowicz, 2019). Whole genome sequencing revealed that within the *Cronobacter* genus, *C. sakazakii* is the only species that can use sialic acid as a carbon source, as it possesses the *nanAKTR* gene cluster (Joseph et al., 2013; Singh et al., 2015). The ability of *C. sakazakii* to metabolize sialic acid may be a factor in PIF survival and infection of the intestines and brain, but further research is required to validate this hypothesis (Jaradat et al., 2014).

### Stress Adaptation

*Cronobacter sakazakii* is a resilient enteropathogen capable of surviving desiccation, heat, and low pH encountered during food manufacturing processes and in the human GI tract (Feeney et al., 2014; Kim et al., 2015; Jameelah et al., 2018; Parra-flores et al., 2018b; Holý et al., 2019). In addition, its resistance against components in the GI tract, including stomach acid and bile liquids, may be a key factor in the development of illnesses across all age groups, especially in infants due to their lower stomach acidity.

*Cronobacter sakazakii* was shown to survive desiccation by accumulating electrolytes to increase osmotic pressure inside the cell, which prevents cellular fluids from leaking out (Aly et al., 2019), but there are other biochemical pathways that play a role in desiccation resistance. For example, the upregulation of *ompW* can contribute to the survival of *C. sakazakii* in dry conditions while promoting biofilm formation (Aly et al., 2019). Bai et al. (2019) reported that the concentration of trehalose increased inside *C. sakazakii* cells experiencing desiccation tolerance and found that the accumulation of trehalose also improved the survival of *C. sakazakii* that were subjected to cold stress. The notion of cross-protection, whereby an acquired tolerance from sublethal stress can cross-protect against other stresses, has been documented in other foodborne pathogens including *E. coli* (Yurtsev et al., 2016), *S*. Typhimurium (Leyer and Johnson, 1993), and *L. monocytogenes* (Begley et al., 2002) but is not well described in *Cronobacter* spp.

*Cronobacter sakazakii* biofilms can form in processing environments on a variety of surfaces including silicon, stainless steel, plastic, silicon, and latex during stressful conditions, such as nutrient limitation or desiccation, which contributes to contamination risk in PIF and other products (Kalyantanda et al., 2015; Singh et al., 2015; Aly et al., 2019). *Cronobacter* spp. have been reported to produce biofilms with the highest cell density among the Enterobacteriaceae (Kalyantanda et al., 2015), further emphasizing the importance of good hygiene and regular cleaning in hospitals and homes. The persistence of mature biofilms in enteral feeding tubes could shed planktonic cells that enter the stomach of infants during feeding, thus contributing to infantile infection (Hurrell et al., 2009; Kalyantanda et al., 2015). Furthermore, *C. sakazakii* and other pathogens can enter a VBNC state during biofilm formation or PIF production (Forsythe, 2014; Jameelah et al., 2018). Biofilms are more resistant to disinfection methods, and some of the biosynthesis pathways that are required to produce biofilms, such as the colanic acid biosynthesis pathway, have been linked to antibiotic, desiccation, heat, and acid resistance.

Infants have a lower gastric acidity and bile salt concentration as compared to adults (Poquet and Wooster, 2016; Neal-Kluever et al., 2019), which may facilitate the survival of foodborne pathogens in the infant GI tract (Tennant et al., 2008; Sistrunk et al., 2016). *Cronobacter sakazakii* cells can survive at pH values as low as 4.5, a bile salt concentration of 5%, a salt concentration up to 10%, temperature as high as 60°C, and over 20days of desiccation (Fakruddin et al., 2014; Jameelah et al., 2018). In fact, the sub-lethal GI conditions in an infant may exacerbate *C. sakazakii* resistance to antimicrobial compounds produced by gut bacteria or probiotics (Hsiao et al., 2010). Furthermore, exposure to sub-lethal acid and thermal conditions may increase the heat resistance of *C. sakazakii* during rehydration of PIF using water <64°C (Yang et al., 2015), which supports the WHO recommendation of rehydrating PIF using water >70°C.

## Biological Models Used To Study *Cronobacter Sakazakii* Pathogenicity

### Cell Culture Models

Pathogens need to adhere to the host cell surfaces to establish infection (Shi et al., 2017). Adherence and invasion of *C. sakazakii* have been primarily studied using cell lines including Caco-2, HT-29, INT-407, and Hep-2 (Feeney et al., 2014; Kim et al., 2015; Ye et al., 2016; Holý et al., 2019). These models are especially important to simulate the attachment and entry of *C. sakazakii* to intestinal cells. Human isolates of *C. sakazakii* have been found to be better at binding to tissue culture cell models as compared to environmental strains (Liu et al., 2012). Following adherence, *C. sakazakii* increased cell permeability and disrupted tight junctions due to NO production, resulting in apoptosis (Liu et al., 2012). *Bacteroides fragilis* ZY-312 and citral were shown to reduce *C. sakazakii* invasion into HT-29 and Caco-2 cell lines, respectively (Shi et al., 2017; Fan et al., 2019). Nair and Venkitanarayanan (2007) showed that OmpA, in addition to the host cytoskeleton, plays an important role in cellular invasion of INT-407. Parra-Flores et al. (2018b) found that *C. sakazakii* isolates from PIF were able to invade Hep-2 cells, possibly due to the presence of outer membrane proteins. It should be noted, however, that experiments with different tissue culture cell lines need to be interpreted with caution, as *C. sakazakii* adheres better to certain cell lines (Holý et al., 2019).

### Animal Models

Animal models, such as rabbit pups, neonatal rats, mice, and nematodes, are infrequently used to study the pathogenesis of *C. sakazakii*, because they cannot accurately replicate the *in vivo* interactions within an infant (Molloy et al., 2010; Hunter and Bean, 2013; Gurien et al., 2018; Fan et al., 2019; Kavita et al., 2020). However, several studies using animal models suggest that probiotic bacteria may be able to reduce the intestinal colonization of *C. sakazakii* and severity of NEC *in vivo* (Gurien et al., 2018; Fan et al., 2019; Kavita et al., 2020). Animal studies are useful to indicate whether prophylactic treatments can be further studied in infants. For example, Gurien et al. (2018) showed that as compared to the control group, pre-treatment of rabbit pups with *Lactococcus lactis* reduced the incidence rate of NEC caused by *C. sakazakii*.

### Gastrointestinal Models

A more advanced approach to study *C. sakazakii* in the GI tract of humans is by using GI models to assess the pathogen’s survival and subsequent environmental changes in the GI tract. The TNO *in vitro* GI model (TIM) can mimic intestinal physiology for nutrition or metabolomic studies (Venema, 2015), whereas the SHIME is a multi-compartment dynamic simulator of the human GI tract that can simulate the stomach, small intestine, and the colon (Van den Abbeele et al., 2010). The TIM has been used to study the survival of *E. coli* or probiotic bacteria (Kheadr et al., 2010; Roussel et al., 2020), but its main advantage is the ability to mimic the luminal conditions in the GI tract through secretions, absorption, and the removal of bioavailable compounds (Minekus, 2015). In contrast, the SHIME system can evaluate interactions between the gut microbiota and/or foodborne pathogens due to its ability to inoculate the colon vessels with fecal samples from different age groups to analyze changes in the gut microbiota or metabolomics based on various experimental parameters (Van de Wiele et al., 2015). However, the SHIME has its own limitations, one of which is the inability to mimic normal peristalsis in the GI tract as it relies on stirrers to mix the GI fluids, so GI interactions and movement dependent on peristalsis may not be accurately portrayed. The TIM and SHIME complement each other to better mimic the complex ecosystem within the GI tract (Roussel et al., 2020), as both models have their unique advantages and disadvantages. Thus far, no studies have been done on the interaction between *C. sakazakii* and an *in vitro* gut model. Given the dynamic nature of an infant gut microbiota and its association with the development of infection, the SHIME can be a useful tool to study the changes in gut microbiota in a simulated infant GI tract when exposed to *C. sakazakii*.

## Probiotics, Prebiotics, and Synbiotics

As current measures have proven insufficient to eliminate *C. sakazakii* contamination and infection, other preventative measures are required. Probiotics, prebiotics, and synbiotics may be promising control measures to inhibit the growth of *C. sakazakii* in the environment and within the infant GI tract. Furthermore, some probiotic strains can have synergistic effects with prebiotic substrates, i.e., synbiotics, as the prebiotic is degraded into antimicrobial metabolites or used to alter the bacterial surface and improve adhesion to the intestinal epithelial layer (Bocchi et al., 2020). Therefore, synbiotics may be better at mitigating the risk of infection due to foodborne pathogens, such as *C. sakazakii*, compared to the independent application of probiotics or prebiotics.

### Probiotics

FAO/WHO developed the first definition for probiotics: “live microorganisms which, when administered in adequate amounts, confer a health benefit on the host” (Food and Agriculture Organization of the United Nations/World Health Organization, 2002). A probiotic strain should have the following characteristics to confer benefits to the host (Food and Agriculture Organization of the United Nations/World Health Organization, 2002; Hill et al., 2014; Nagpal et al., 2018; Binda et al., 2020; Kaur et al., 2021; Rastogi et al., 2021): (1) isolated from a human, (2) lacking in putative virulence genes, (3) sensitive to common antibiotics, (4) tolerant to GI conditions, (5) catalase-negative, (6) able to adhere to the intestinal epithelial membrane, (7) able to compete with native gut microbiota, and (8) able to directly or indirectly inhibit the growth and colonization of potentially pathogenic bacteria. Research into probiotics has quickly expanded, with over 32,000 articles being published between 2002 and 2021 using the term “probiotic,” but proving the health benefits of probiotics, such as improving gut health after the consumption of probiotics, is challenging. Therefore, governments have taken different regulatory approaches on food products containing probiotics (Table 4).

**Table 4:** Comparison of probiotic regulations between countries and regions.

Country/region	Accreditation body	Regulation	References
Canada	Health Canada	Validating health claims on strain-specific evidence, clear and specific statements on the probiotic benefits, and documentation on the strain added to the food product.	Health Canada, 2009
United States	U.S. Food & Drug Administration (FDA)	Regulated as dietary supplements, foods, or drugs depending on the product’s intended use. Products containing probiotics are subject to additional regulations based on their classification (i.e., supplements or foods).	U.S. Food and Drug Administration, 2006
European Union	European Food Safety Authority	Based on Regulation N° 1924/2006, probiotics are classified as a health claim to imply a health benefit once ingested.	European Commission, 2007
Australia/New Zealand	Food Standards Australia and New Zealand (FSANZ)	Products containing probiotics are assessed on a case-by-case basis based on Standards 1.2.7 regulation. Information required for risk assessment includes composition, safety, health claim, and history of use in other countries.	Food Standards Australia and New Zealand, 2013; Food Standards Australia and New Zealand, 2021
Asia-Pacific	Ministry of Health, Labor and Welfare (Japan) and State Administration for Public Regulation (China)	In Japan, food products containing probiotics must include health claims such as improving GI health. Probiotics included in foods sold in China require live microbes as the main functional ingredient and must provide information on the strain and its associated health benefits.	Chemical Inspection and Regulation Service, 2019; Iwatani and Yamamoto, 2019
Brazil	Agência Nacional de Vigilância Sanitária	Strains are approved on a case-by-case basis and must demonstrate safety and health benefits based on Resolution N° 18/1999.	International Probiotics Association, 2017

*Bifidobacterium* and *Lactobacillus* genera are the earliest examples of probiotics included in food products as they are Generally Recognized as Safe and can impart general health benefits such as supporting a healthy GI tract and immune system (Food and Agriculture Organization of the United Nations/World Health Organization, 2002; Hill et al., 2014; Canadian Food Inspection Agency, 2019). PIF can also be supplemented with probiotics, however, their application and scope of use in Canada is limited. Even among *Bifidobacterium* and *Lactobacillus* spp., only *Bifidobacterium animalis* subsp. *lactis* (BB-12) and *Lactobacillus rhamnosus* GG (LGG) are commercially available in PIF sold in Canada (Alberta Health Services, 2017) at a dose of approximately 7–8 log CFU/100ml. The approval of BB-12 and LGG may be due to the vast amounts of research that has been done on these specific strains to validate their safety and potential ability to exert health benefits on the host (Jungersen et al., 2014; Taipale et al., 2016; Flach et al., 2018; Capurso, 2019; Szajewska and Hojsak, 2020). However, the probiotics have not yet been approved by the Health Canada or the CFIA. Canadian probiotic supplements are designed for infants and contain either a single strain or a cocktail of *Bifidobacterium* and *Lactobacillus* spp. (AEProbio, 2020). The probiotic supplements have been reported to reduce the occurrence and mortality of NEC in premature and low-birthweight infants by approximately 4–12% (Hunter et al., 2012; Janvier et al., 2014). While the benefits of probiotics to overall health continue to be studied, research has shown several mechanisms by which probiotics can inhibit the growth of foodborne pathogens.

#### Antimicrobial Capabilities of Probiotics

Probiotics can indirectly mitigate human infections by (i) enhancing epithelial barrier physiology; (ii) inhibiting virulence gene expression; (iii) enhancing the host immune system; (iv) directly attacking pathogens by producing metabolites; (v) competing with the pathogens for nutrients, and (vi) adhering to the surface of GI cells, thus providing competition for attachment (Tomar et al., 2015). In the large intestine, they primarily produce organic acids due to the absence of oxygen (Dittoe et al., 2018). In the stomach and small intestine, the minute concentration of oxygen allows probiotics to produce H_2_O_2_ in enough quantities to directly inhibit pathogens; however, low concentrations of H_2_O_2_ in the anaerobic environment of the large intestine can only reduce pathogen invasion rather than exerting a bactericidal effect (Knaus et al., 2017). Different probiotic strains are very efficient in producing different compounds to inhibit pathogens. For example, *Lactobacillus casei* and *L. acidophilus* were found to produce bacteriocins that could inhibit the growth of *C. sakazakii* in reconstituted PIF (Awaisheh et al., 2013) and *Bifidobacterium* spp. produce organic acids, such as acetic acid, lactic acid, and formic acid, that inhibit other pathogens (Makras and De Vuyst, 2006).

The effect of probiotics on the gene expression of *C. sakazakii* has not been studied thus far, but previous research has shown that probiotics can downregulate virulence gene expression of other enteropathogens such as *E*. *coli* O157:H7 and *Clostridium difficile* (Yun et al., 2014; Mohsin et al., 2015). *Escherichia coli* Nissle 1917, a known probiotic strain, was able to control the Shiga-toxin gene expression of enterohemorrhagic *E. coli* O104:H4 and O157:H7 *in vitro* by downregulating *stx2* mRNA transcription (Mohsin et al., 2015). *Bifidobacterium longum* and other probiotic strains have also been shown to downregulate Shiga toxin gene expression *in vitro* and *in vivo* (Carey et al., 2008; Cordonnier et al., 2016). It is possible that the downregulation of Shiga toxin gene expression was due to environmental stresses created by probiotics through the inhibition of the autoinducer-2 or a similar autoinducer system (Park et al., 2017). While the inhibitory effects of probiotics on enteropathogens have been documented, the mechanisms by which they exert their protective effects require further investigation.

### Postbiotics

Probiotics are strictly regulated, and concise guidelines for their application in clinical settings (e.g., to reduce NEC) are lacking, thus creating an opportunity to study the application of their by-products to achieve the same beneficial effects (Piqué et al., 2019). Postbiotics have been defined as a “preparation of inanimate microorganisms and/or their components that confers a health benefit on the host,” thereby containing inactivated microbial cells that may contain metabolites or cellular components to exert said health benefit (Salminen et al., 2021). Postbiotics can originate from a probiotic strain and can be composed of the cell-free supernatant (CFS) consisting of bacteriocins, organic acids, H_2_O_2_, and other compounds that can inhibit bacterial cells or biofilm formation (Campana et al., 2019; Jamwal et al., 2019; Piqué et al., 2019; Nataraj et al., 2020). Awaisheh et al. (2013) showed that *L. casei* and *L. acidophilus* CFS inhibited the growth of *C. sakazakii* on agar media and a liquid rehydrated infant formula model, while Lin and Pan (2019) demonstrated that *Lactobacillus plantarum* NTU 102 CFS contained an active antimicrobial compound, named as LBP102, which was able to inhibit *C. sakazakii in vitro*. Other *Lactobacillus* spp. CFS has been shown to weaken the membrane integrity and disrupt the biofilm formation of *C. sakazakii in vitro* (Charchoghlyan et al., 2016; Campana et al., 2019; Jamwal et al., 2019), indicating the potential for a multi-faceted application of probiotic strains CFS. Several antimicrobial compounds in CFS were heat-resistant, which may be useful as food preservatives for manufacturing foods that are subject to high heat during processing, e.g., spray drying of PIF (Jamwal et al., 2019; Lin and Pan, 2019). Specifically, CFS can contain postbiotic metabolites, such as SCFA, which will be discussed later in this review, and bacteriocins that have antimicrobial properties against pathogenic bacteria and viruses including *E. coli*, *C. difficile*, *C. sakazakii*, *Salmonella* spp., influenza, and rotavirus (Mantziari et al., 2020; Nataraj et al., 2020). As postbiotics are composed of the bioactive components of an inactivated probiotic, their mechanisms to exert a health benefit on the host are similar to their live counterpart. Postbiotics can modulate the immune system through the production of toll-like receptors, reduce the adhesion of pathogens to the gut epithelium by competitive exclusion, or inhibit the growth of pathogens through bacteriocins (Dunand et al., 2019; Mantziari et al., 2020; Mayorgas et al., 2021). As shown in Table 5, there have been other successful applications of probiotic strains and their CFS in inhibiting *C. sakazakii*.

**Table 5:** Summary of probiotic strains reported to decrease *C. sakazakii* infections.

Probiotic	Application	Age group	Outcome	References
*Lactobacillus reuteri* DSM 17938	*In vivo*	Very low birth weight (<1,500g birth weight)	Reduced incidence of NEC	Hunter et al., 2012
*L. casei* or *L. acidophilus* CFS and live cells	*In vitro* and reconstituted PIF		Reduced viability of *C. sakazakii* in both treatments and applications	Awaisheh et al., 2013
Cocktail of *Bifidobacterium* spp. and LGG	*In vivo*	Preterm infants (<37weeks gestational age)	Reduced frequency in neonatal intensive care unit	Janvier et al., 2014
*Bifidobacterium infantis* CFS	*In vivo*; mice model		Protection against *C. sakazakii* induced ileal inflammation	Weng et al., 2014
*L. acidophilus* strain Narine CFS	*In vitro* and reconstituted PIF		Inhibition and damaged cells of *C. sakazakii*	Charchoghlyan et al., 2016
*Bacteroides fragilis* ZY-312, next-generation probiotic candidate	*In vitro*		Inhibited *C. sakazakii* invasion and regulates cell apoptosis	Fan et al., 2019
*L. rhamnosus* or *L. acidophilus* CFS	*In vitro*		Inhibition and damaged cells of *C. sakazakii*	Campana et al., 2019
Various *Lactobacillus* spp. CFS	*In vitro*		Inhibited *C. sakazakii* biofilm formation	Jamwal et al., 2019

### Prebiotics

Prebiotics were originally defined as non-digestible carbohydrates that are metabolized by the commensal gut microbiota in the colon (Bezkorovainy, 2001). Recently, the definition of prebiotics has been modified to include substrates used by the GI microbiota that confer health benefits to the host, extending the substrate candidates to include more than just carbohydrates, e.g., phenols and phytochemicals (Gibson et al., 2017). To exert health benefits, prebiotics should resist host digestion, be fermented by gut microbes, and selectively promote the growth of beneficial bacteria, e.g., *Lactobacillus* and *Bifidobacterium* spp. (Gibson et al., 2017). In infants, prebiotics can also improve nutrient absorption, decrease the translocation of pathogenic bacteria through gut maturation, and enhance overall GI health (Davis et al., 2017). Common prebiotics included in PIF sold in Canada typically contain GOS, inulin, or polydextrose (PDX) to provide the aforementioned health benefits to infants (Davis et al., 2017). Goat and bovine-milk derived oligosaccharides are currently being investigated for their prebiotic properties and their beneficial effects as part of infant formula (Simeoni et al., 2016; Jakobsen et al., 2019; Leong et al., 2019; Gallier et al., 2020). Goat and cow milk were found to exert bifidogenic effects similar to those from human milk (Simeoni et al., 2016; Jakobsen et al., 2019; Gallier et al., 2020). Additionally, goat milk oligosaccharides reduced the adhesion of *E. coli* NCTC 10418 and *S*. Typhimurium to Caco-2 cells (Leong et al., 2019). *Bifidobacterium infantis* was found to metabolize HMOs, but the enzymes used to break down HMOs are not present in other *Bifidobacterium* spp. more commonly found in adults such as *B. longum* and *B. lactis* (Gibson et al., 2017). Furthermore, prebiotics were shown to be heat-resistant (Huebner et al., 2008; Moore, 2011; Vega and Zuniga-Hansen, 2015), thus providing important nutrients for gut maturation even in heat-treated products, e.g., pasteurized donor breast milk (Mueller et al., 2015).

Prebiotics, including breast milk HMOs, are capable of preventing pathogens and their toxins from adhering to intestinal cells by acting as molecular decoys in the GI tract, indicating why early breast-feeding is a key factor in developing a healthy gut microbiome (Morrow et al., 2005; Le Doare et al., 2018). HMOs are the first prebiotics consumed by infants, comprising approximately 1.1% of breast milk, and are broken down by certain species of bifidobacteria such as *Bifidobacterium longum* subsp. *infantis*, *B. longum* subsp. *longum*, *B. bifidum*, and *B. breve* (Thomson et al., 2018; James et al., 2019; Lawson et al., 2020). However, other species of *Bifidobacterium* and other gut microbiota can cross-feed on different metabolites, including acetate and fucose, released from HMO degradation (Boehm et al., 2005; Johnson-Henry et al., 2016; Thomson et al., 2018; James et al., 2019; Lawson et al., 2020). Breast feeding is typically done for the first 6months of life (Robertson et al., 2019) and during this time, *B. infantis* is likely to dominate the infant gut microbiota due to the abundance of HMOs (Vatanen et al., 2019). The abundance of *B. infantis* and other *Bifidobacterium* spp. helps to develop a healthy infant gut microbiome in addition to modulating the immune system (Johnson-Henry et al., 2016; Vatanen et al., 2019). Sialic acid on HMOs may play a role in reducing the risk of NEC pathogenesis through complex interactions with the infant immune system, but the mechanisms are unclear (Bode, 2018). One possible method is through the degradation of sialic acid by bifidobacteria due to the production of sialidases located on the *nan-nag* locus (Egan et al., 2014; Lawson et al., 2020; Wu et al., 2021), which results in competition for nutrients with opportunistic pathogens, such as *C. sakazakii* and *C. difficile*, that can also metabolize sialic acid in the colon (Egan et al., 2014).

Prebiotics also can prevent pathogenic colonization and growth, with the latter effect being due to gut microbe metabolism. GOS, for example, contains structures similar to the microvilli present in the colon, and can interfere with pathogen specific receptors and subsequent binding (Tomar et al., 2015). The effects of prebiotics on *C. sakazakii* and NEC are not well-known, but GOS and PDX have been shown to reduce the adherence of *C. sakazakii* to the HEp-2 cell line by 42–71% when used separately or combined (Quintero et al., 2011). However, the concentrations of GOS and PDX used in the latter study were higher than those present in PIF (Kent et al., 2015). Srinivasjois et al. (2013) showed that supplementing infant formula with different prebiotic oligosaccharides significantly improved the growth of bifidobacteria but did not decrease the risk of NEC or other illnesses associated with *C. sakazakii* infection in preterm infants. Additionally, prebiotics can affect *C. difficile* toxicity differently as demonstrated by analyzing changes in HT-29 cell attachment and growth, thus indicating that carbon source can play a factor in pathogen inhibition (Valdés-Varela et al., 2016). In addition to indirectly decreasing the risk of infection by reducing pathogen adhesion, prebiotics can also provide benefits to the host once metabolized by the gut microbiota.

### Role of SCFA in Inhibiting Pathogens and Promoting the Development of a Healthy Gut

Some SCFAs, such as butyrate and propionate, are commonly produced by commensal bacteria in the GI tract and have been extensively studied (Nagpal et al., 2018). Reduced levels of SCFA in the GI tract have been linked to various illnesses including obesity, type II diabetes, and autoimmune diseases in adults, but their association with metabolic and immune conditions in infants is not well studied (Nagpal et al., 2018; Differding et al., 2020). In the human intestinal tract, butyrate and propionate regulate inflammation, epithelial barriers, and intestinal motility and may be able to reduce the risk of various medical conditions such as colitis and intestinal inflammation (Canani et al., 2011; Engels et al., 2016). However, it should be noted that acetate and lactate both have an important role to play as precursors for the production of butyrate and propionate (Venegas et al., 2019). Some gut bacteria, such as *Eubacterium hallii*, can produce SCFAs from metabolites created by bifidobacterial fermentation, which can play a role in inhibiting pathogens in the gut (Engels et al., 2016; Schwab et al., 2017). The acid conjugates of SCFA, such as acetic acid, can diffuse freely in the cytoplasm of pathogens, and this may ultimately compromise metabolic functions and disturb cell physiology (Sun and O’Riordan, 2013).

In moderate quantities, SCFAs can regulate conditions associated with dysbiosis in infants, e.g., maintain the gut barrier, and reduce inflammation caused by the onset of NEC (Differding et al., 2020; Zheng et al., 2020), but higher concentrations of SCFA may be toxic to infants. The normal concentration of total SCFA in humans range from 20 to 140 mM in the large intestine, but concentrations >300mM may be fatal to infants (Nafday et al., 2005). While the mechanism of SCFA toxicity requires further study, excess quantities of SCFA can cause NEC, as it appears that they can reduce carbohydrate absorption and GI motility, which damages gut mucosal integrity (Lin, 2004) – the latter effect can increase SCFA accumulation and exacerbate intestinal injury (Roy et al., 2018). The potential risk of SCFA toxicity indicates that any underlying health conditions, such as poor digestion in infants, should be considered prior to the application of prebiotics. Conversely, high levels of SCFA in fecal samples may indicate lower microbial diversity in the gut and higher gut permeability, both of which are associated with several health complications including obesity, glycemia, and hypertension (De la Cuesta-Zuluaga et al., 2019). However, there is insufficient evidence on the role of a specific SCFA to maintain a healthy gut, as each metabolite is part of a complex gut ecosystem.

Prebiotics and SCFA can benefit host health, but their success is limited to the gut microbiota present in the host. If an individual lacks the microbes necessary to metabolize the prebiotics consumed, possibly due to dysbiosis, it is unlikely that the prebiotic will confer health benefits to the host (Gibson et al., 2017). In this scenario, the individual may benefit instead from a synbiotic supplementation.

### Synbiotics

A synbiotic is defined as “a mixture comprising live microorganisms and substrate(s) selectively utilized by host microorganisms that confers a health benefit on the host” (Swanson et al., 2020). During its conception, synbiotic supplementation was thought to prolong the shelf-life of foods containing live microbes, increase the survival of probiotics, and stimulate metabolism of the gut microbiota (Gibson and Roberfroid, 1995). Other beneficial claims of synbiotics include larger populations of *Lactobacillus* and *Bifidobacterium* spp. in the GI tract, improved immune system function, and reduced risk of bacterial infection in higher risk patients (Pandey et al., 2015). Since the gut microbiota and probiotics respond differently to prebiotics, compatibility is an important factor in formulating synbiotics.

When *Lactobacillus* strains were grown in the presence of different prebiotics, such as inulin, fructooligosaccharide (FOS), and lactulose, *L. plantarum* demonstrated the most rapid growth with inulin (Nagpal and Kaur, 2011). Differences in oligosaccharide fermentation were shown to exist between *L. lactis* subsp. *lactis* strains, as one strain possesses specific enzymes for the transport and hydrolysis of palatinose, yet another strain was unable to ferment palatinose (Pranckute et al., 2014). In another study, *Bifidobacterium* spp. were demonstrated to be better at fermenting glucooligosaccharides as compared to *Lactobacillus* spp. possibly due to the presence of numerous genes for carbohydrate metabolism (Grimoud et al., 2010). Similarly, β-galactosidases in bifidobacteria are better at hydrolyzing β(1→6) and β(1→3) linked GOS as compared to lactobacilli (Kittibunchakul et al., 2018). To summarize, different bacterial species and strains within a species produce unique enzymes that make them more compatible with certain prebiotics (Pranckute et al., 2014).

The use of synbiotic supplementation in infants has shown encouraging results (Chua et al., 2017; Vandenplas et al., 2017), but these studies are rarely conducted possibly due to ethical concerns. Ingestion of a synbiotic supplement in preterm infants can decrease the risk of disease by increasing the populations of beneficial gut microbiota and/or reducing pathogen adhesion (Luoto et al., 2014; Kent et al., 2015). Although more studies are needed to validate the findings, synbiotic supplementation in premature infants may have additional benefits, as randomized clinical trials have shown a decrease in NEC cases relative to the control group (Vongbhavit and Underwood, 2016).

Breast milk is known to be the ideal synbiotic as it not only contains beneficial bacteria, such as *Lactobacillus*, *Bifidobacterium*, *Rothia*, *Enterococcus*, and *Veillonella* spp., and prebiotic HMOs, but also provides immune factors to stimulate the immunological development of infants (Bertelsen et al., 2016; Le Doare et al., 2018; Robertson et al., 2019; Fehr et al., 2020; Lyons et al., 2020). Breast milk is associated with an increase in the levels of *Bifidobacterium* and, as the gut matures, *Akkermansia muciniphila*, *Faecalibacterium prausnitzii* and *Lactobacillus* spp. among other obligate anaerobes, also increase in numbers (Robertson et al., 2019). *Akkermansia muciniphila* and *F. prausnitzii* have been suggested to be candidates for next-generation probiotics due to their association with a healthy gut and immune system, and potential to reduce NEC (Underwood, 2017; Maftei, 2019).

To the best of our knowledge, there has not been any research on the effect of synbiotics on *C. sakazakii*, although several studies have shown reduced morbidity or mortality of NEC after synbiotic application (Dilli et al., 2015; Sreenivasa et al., 2015; Nandhini et al., 2016; Pehlevan et al., 2020), and other studies have shown the beneficial effects of synbiotics on other foodborne pathogens such as *Salmonella enterica*, *Shigella sonnei*, and enteropathogenic and enterohemorrhagic *E. coli* (Likotrafiti et al., 2016; Kusmivati and Wahyuningsih, 2018; Shanmugasundaram et al., 2019; Tabashsum et al., 2019; Piatek et al., 2020). The antimicrobial potential of synbiotics is likely dependent on the pathogen-synbiotic combination as *E. coli* and *Campylobacter jejuni* were inhibited by synbiotics when cultured in a multi-batch culture system containing a synbiotic with a pathogen, but in a single continuous culture system only *C. jejuni* was inhibited (Fooks and Gibson, 2003). The excess carbon source in the continuous culture prevented nutrient competition, suggesting that antimicrobial compounds, i.e., SCFA, were produced, and that inhibition efficacy can differ depending on the pathogen-probiotic combination (Fooks and Gibson, 2003). Valdés-Varela et al. (2016) further expanded on the species dependency premise to include substrate dependency. For example, *B. longum* and *B. breve*, instead of *B. bifidum* or *B. animalis* and in the presence of FOS instead of inulin, inhibited *C. difficile* (Valdés-Varela et al., 2016). Another study noted that *B. longum* in the presence of the prebiotic isomaltooligosaccharides inhibited *E. coli* O157:H7 and *E. coli* O86 better than *Lactobacillus fermentum* with FOS (Likotrafiti et al., 2016). The interaction between synbiotics and pathogens is complex and requires sophisticated models to better determine their efficacy as a potential treatment for the control of foodborne pathogens.

## Conclusion

*Cronobacter* can cause severe illness in infants, especially those who are premature or of low-birthweight. Due to their ubiquity and resilience, current preventative measures to control invasive *Cronobacter* infections during early infancy would benefit from alternative approaches to minimize any potential risks to infants. Probiotics, prebiotics, and synbiotics are well-placed to play a major role in this regard and can potentially act to prevent intestinal colonization of infants by pathogenic *Cronobacter* spp. or other enteropathogens. However, current research on their efficacy to inactivate or inhibit the growth of pathogenic *Cronobacter* spp. is limited. While there is evidence that the consumption of probiotics, prebiotics, and synbiotics can reduce the risk of infection through competitive exclusion, the production of antimicrobial compounds, or competition for nutrients, further research is required to validate these hypotheses using more complex *in vitro* models and *in vivo* studies. The development of novel models that simulate the human gut provides an exciting opportunity to study in real-time the interactions between *Cronobacter* spp. and the native infant gut microbiota *in vitro*.

## Author Contributions

AK researched, wrote the topics within the manuscript, and revised the manuscript. VP, JF, and LG revised the manuscript including grammar, syntax, and information gap. All authors contributed to the article and approved the submitted version.

## Funding

We would like to acknowledge the financial support from the Canada First Research Excellence Fund (CFREF) through the Food from Thought initiative. Grant #499114.

## Conflict of Interest

The authors declare that the research was conducted in the absence of any commercial or financial relationships that could be construed as a potential conflict of interest.

## Publisher’s Note

All claims expressed in this article are solely those of the authors and do not necessarily represent those of their affiliated organizations, or those of the publisher, the editors and the reviewers. Any product that may be evaluated in this article, or claim that may be made by its manufacturer, is not guaranteed or endorsed by the publisher.

## Footnotes

^1^ http://tools.fstools.org/esakmodel/ESAKRAModelWizard.aspx
